# Determination of the Entire Stent Surface Area by a New Analytical Method

**DOI:** 10.3390/ma13245633

**Published:** 2020-12-10

**Authors:** Muhammad Saqib, Ricardo Bernhardt, Markus Kästner, Natalia Beshchasna, Gianaurelio Cuniberti, Jörg Opitz

**Affiliations:** 1 Fraunhofer Institute for Ceramic Technologies and Systems IKTS, Maria-Reiche-Strasse 2, 01109 Dresden, Germany; 2 Faculty of Mechanical Science and Engineering, Institute of Materials Science and Max Bergmann Center of Biomaterials, Technische Universität Dresden, 01062 Dresden, Germany; bernhardt@ipfdd.de; 3 Leibniz Institute of Polymer Research Dresden, 01069 Dresden, Germany; 4 Faculty of Mechanical Science and Engineering, Institute of Solid Mechanics, Technische Universität Dresden, 01062 Dresden, Germany; markus.kaestner@tu-dresden.de; 5 Dresden Center for Computational Materials Science, Technische Universität Dresden, 01062 Dresden, Germany; 6 Center for Advancing Electronics Dresden, Technische Universität Dresden, 01062 Dresden, Germany

**Keywords:** stents, surface area, micro-CT, degradation, corrosion, mass loss, quantitative corrosion test

## Abstract

Stenting is a widely used treatment procedure for coronary artery disease around the world. Stents have a complex geometry, which makes the characterization of their corrosion difficult due to the absence of a mathematical model to calculate the entire stent surface area (ESSA). Therefore, corrosion experiments with stents are mostly based on qualitative analysis. Additionally, the quantitative analysis of corrosion is conducted with simpler samples made of stent material instead of stents, in most cases. At present, several methods are available to calculate the stent outer surface area (SOSA), whereas no model exists for the calculation of the ESSA. This paper presents a novel mathematical model for the calculation of the ESSA using the SOSA as one of the main parameters. The ESSA of seven magnesium alloy stents (MeKo Laser Material Processing GmbH, Sarstedt, Germany) were calculated using the developed model. The calculated SOSA and ESSA for all stents are 33.34%(±0.26%) and 111.86 mm (±0.85 mm), respectively. The model is validated by micro-computed tomography (micro-CT), with a difference of 12.34% (±0.46%). The value of corrosion rates calculated using the ESSA computed with the developed model will be 12.34% (±0.46%) less than that of using ESSA obtained by micro-CT.

## 1. Introduction

The stenting of arteries is the most common treatment procedure for coronary artery disease [1]. Undeniably, stenting has been a very fast-growing field since its beginning [2,3]. Due to the rapid advancements in stent technology, different kinds of stents are being implanted for the treatment of blocked arteries—for instance, bare-metal stents, drug-eluting stents, bioresorbable stents, coated stents, dual therapy stents, and bioengineered stents [4].

To overcome the existing clinical complications [5,6] due to the complex interaction of stents in a physiological environment, stents need to be further developed. For this purpose, new stent technologies [7,8,9,10] are manufactured to improve the clinical outcomes. However, for the successful development of stent technology, innovative in vitro approaches for stent testing are important.

The effective lifetime of any implant material highly depends on its corrosion properties. In vitro corrosion tests include static immersion tests, fluid-dynamic tests, and electrochemical tests. For the quantitative analysis of corrosion, the surface area exposed to the corrosive medium has to be defined [11,12].

For instance, the corrosion rate in static immersion and fluid dynamic tests is calculated by Equation (1) [12]. For the calculation of corrosion rates using electrochemical tests, Equation (2) [11] is used.

(1) $$CR=\frac{W\times 8.76\;\times 10^{4}}{At\rho }$$

where *CR* is the corrosion rate in mm yr^−1^, *W* is the mass loss in g, *A* is the original surface area exposed to the corrosive media (simulated body fluids) in cm^2^, *t* is the exposure time in h, and *ρ* is the standard density in g·cm^−3^.

(2) $$CR=K_{1}\frac{i_{corr}}{\rho }\;EW$$

where *CR* is the corrosion rate in mm yr^−1^, *K*_1_ is the constant and its value is 3.27 × 10^−3^ mm·g·µA^−1·^cm^−1·^yr^−1^, *i*_corr_ is the corrosion current density in µA·cm^−2^ (it is the amount of current flow per unit exposed surface area of the sample), *EW* is the equivalent weight in g·eq^−1^, and *ρ* is the standard density in g·cm^−3^.

In both the aforementioned equations, the exposed surface area is essential for the calculation of the corrosion rate. This is simple in the case of regular-shaped geometrical samples—e.g., rounded and square samples, wire, or hollow tubes.

Stents have a very complex geometry, which makes it difficult to calculate the exposed surface area. Therefore, in the case of stents, quantitative corrosion tests cannot be performed due to the lack of methods for the calculation of the surface area. For this reason, simpler geometrically shaped samples are used as a reference to predict the corrosion behavior of stents inside the in vivo environment [13,14,15].

In the last two decades, plenty of studies have been conducted on stent mechanical testing, fatigue analysis, etc. [16,17]. However, in the case of corrosion testing with stents, only a limited number of studies have been conducted. For instance, there are studies focused on the corrosion behavior of stents [18,19,20] which do not use quantitative analysis or use only the outer or upper stent surface [19]. The aforementioned work, by Hertel et al. 2016 [18], includes only a small part of the outer side of the stent struts on various regions exposed to the corrosive medium and conducted electrochemical corrosion tests on bare-metal stents. The study does not consider the influence of geometry on the corrosion behavior of stents. The variability of coating thickness on different surfaces of a coated metallic stent may also affect the results. Furthermore, this technique is also not applicable to other kinds of degradation tests and corrosion tests—for example, immersion and fluid dynamic tests.

Several techniques have been developed to investigate the stent outer surface area (SOSA) or metallic surface area (MSA) of the stent [21,22]. The SOSA or MSA is the ratio of the outer stent surface which is in contact with the vessel. It includes only the outer side of the strut and does not represent the entire stent surface area. In our previous work [23], we presented the application of micro-CT for the determination of the total or entire stent surface area.

Although micro-CT is a well-established non-destructive method, it can be cost and time intensive and is therefore not a standard procedure for stent surface quantification within a common laboratory. As a consequence, a new characterization method is needed for stent surface quantification. Subsequently, to enable the corrosion experiments directly with stents instead of using only a small part to better replicate the degradation behavior inside the artery, this study aimed to develop a non-destructive method for the entire stent surface area calculation with the help of the mathematical model.

In this paper, we present a novel method to determine the entire stent surface area by a developed formula validated with the help of the micro-CT analysis [23]. The presented model was applied to the Mg alloy stent samples. Both calculated and micro-CT surface area values have a maximum deviation of about 12.92%.

The obtained results form the basis of further research and development for stent corrosion testing. By using this model, experiments can be directly conducted on stents and their corrosion behavior can be better understood.

## 2. Stent Outer Surface Area (SOSA)

As mentioned above, this surface area only covers the outer surface of the stent. It can also be expressed as the metallic surface area (MSA) of the stent or the metal to artery ratio (MAR). SOSA is a ratio between the stent outer surface and the covered vessel inner surface area (Equation (3)). In the ISO standard 25539-2 [24], the SOSA has been defined as the contact area between the stent and the vessel. The SOSA is inversely proportional to the stent-free surface area (SFSA), which is a percentage of the surface area of the cylinder formed by the implant frame which is not covered by implant material [24]. In practical applications, SOSA or MSA are preferred by manufacturers to the SFSA.

(3) $$SOSA=\frac{A_{stent}}{A_{vessel}}\;\cdot \;100\;\left(\%\right)$$

where *A_stent_*: outer surface of the stent; *A_vessel_*: surface of the covered vessel.

Karoly et al. [21] and Kovacs et al. [22] developed and presented the following three methods for the calculation of the SOSA.

### 2.1. Calculation Method

Karoly et al. [21] developed this method using various stent values, such as stent diameter, length, and strut thickness. The ratio of the mass of the hollow tube (same material properties and wall thickness to those of the stent) to the mass of the stent is considered a metallic surface area. Equation (4) shows the mass of the ideal tube which has a 100% MSA value given as:

(4) $$m_{tube}=d\pi ls\rho$$

where *m_tube_*: mass of the tube; *d*: stent outer diameter; *l*: stent length; *s*: tube’s wall thickness (strut thickness of the stent); *ρ*: density of the stent.

The metallic surface area can be calculated using Equation (5) with the ratio of the stent mass (*m_stent_*) and the calculated mass of the ideal tube:

(5) $$\mathrm{SOSA}=\frac{m_{stent}}{m_{tube}}$$

### 2.2. Manual Method

This method is based on manual stereomicroscopy. The stents were rotated by increments of 15° from the starting 0° position to 360°, and high-resolution digital images were taken of each segment of the stents’ mantle with a Olympus SZX16 stereomicroscope (Olympus Corporation, Shinjuku, Tokyo, Japan) fitted to a precision rotary unit. The pictures were then joined and examined with JMicroVision image analysis software.

### 2.3. Automatic Method

In this method, the stents were pulled up to a shaft connected to a motor. The stents were rotated during the scanning process to produce one whole picture of the stent pattern. In the scanning platform, the rotational speeds and direction can be changed.

## 3. Entire Stent Surface Area (ESSA)

**Figure 1:**
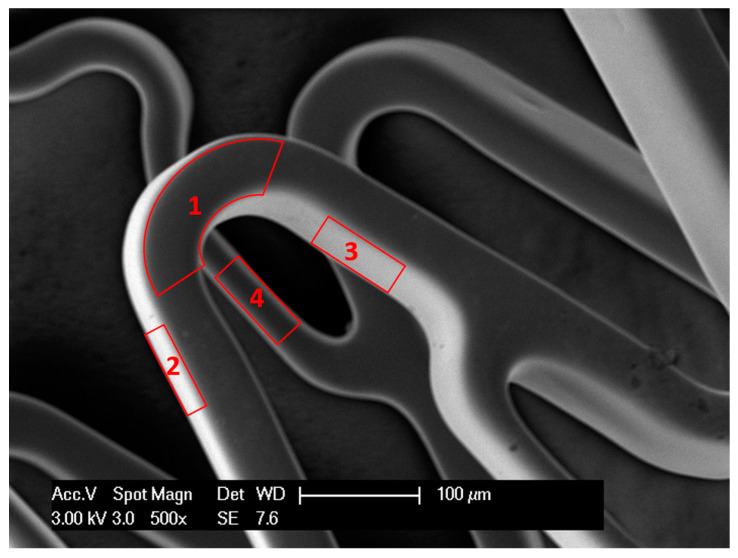
Scanning electron microscopy (SEM) image of a stent showing 4 surfaces of stent wire: upper or outer surface (1), bottom or inner surface (4), right and left surfaces (2,3).

**Figure 2:**
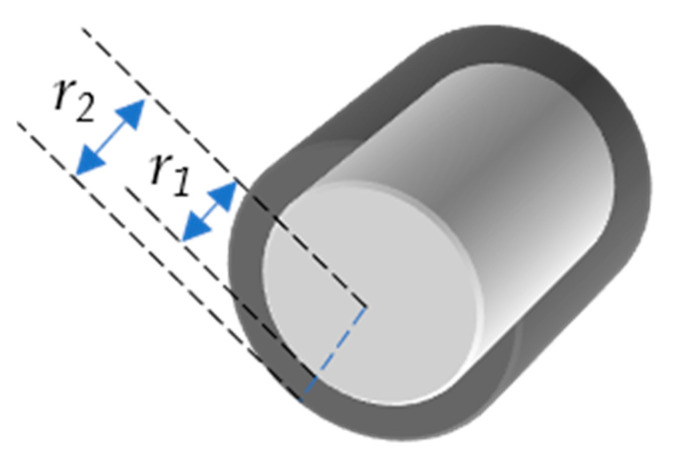
Hollow cylinder with inner radius r_1_ and outer radius r_2_.

The ESSA covers all surfaces of the stent and is the sum of all of the surface areas. In particular, the entire or total stent surface area is the sum of the surface area of all four sides (Figure 1) of all crowns of the stent (Equation (6)). For simplicity, we use ESSA as *A* and SOSA as *A_out_*. Consider stent as a hollow cylinder, where *r*_1_ is the radius of that cylinder from the outer surface, whereas *r*_2_ is the radius from the inner surface (Figure 2). The inner and outer surface areas (*S*_1_ and *S*_2_) of this hollow cylinder can be expressed as Equations (7) and (8).

(6) $$A=\;A_{1}+A_{2}+A_{3}+A_{4}$$

(7) $$S_{1}=2\pi r_{1\;}l$$

(8) $$S_{2}=2\pi r_{2\;}l$$

Since *S_1_* and *S_2_* are the surface areas of the cylinder, to get the surface area of the stent we have to multiply it with the SOSA. The surface areas of all sides will be:

(9) $$A_{1}=\;S_{1}A_{out}$$

(10) $$A_{2}=\;S_{2}A_{out}$$

(11) $$A_{3}=\;cS_{1}A_{out}=\;cA_{1}$$

(12) $$A_{4}=\;cS_{2}A_{out}=\;cA_{2}$$

where *c* is the ratio between the strut thickness (*s*) and the average width of the outer surface of the stent wire (*w*).

(13) $$c=\frac{s}{w}$$

Substituting the values of *A*_1_, *A*_2_, *A*_3_, and *A*_4_ from Equations (9)–(12) in Equation (6), we get:

(14) $$A=\;S_{1}A_{out}+S_{2}A_{out}+cS_{1}A_{out}+cS_{2}A_{out}$$

(15) $$A=A_{out}\cdot \left(1+c\right)\left(S_{1}+S_{2}\right)$$

(16) $$A=2\pi l\cdot A_{out}\cdot \left(1+c\right)\left(r_{1}+r_{2}\right)$$

This is the standard equation to calculate the stent total surface area. Variation in widths in the stent outer surface area may increase the error. The less the variation in widths of outer surface, the more accurate the calculation of the surface area will be.

### 3.1. Value of c

**Figure 3:**
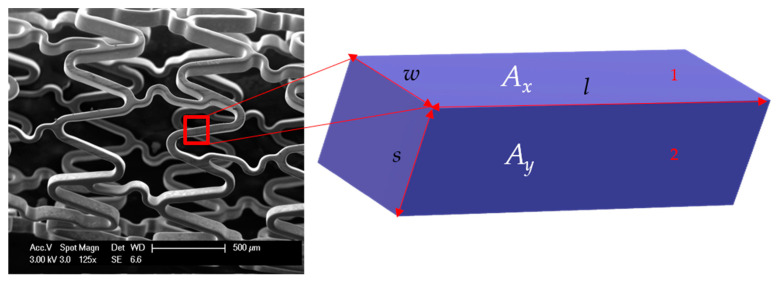
Part of stent wire with length *l*, width of outer surface *w*, and strut thickness *s*. The three sides of this rectangular block are *s*, *l*, and *w*. *A_x_* is the area of surface 1 with sides *w* and *l*. *A_y_* is the area of surface 2 with sides *s* and *l*.

Let *A_x_* and *A_y_* be the areas of surfaces 1 and 2, respectively (Figure 3). Then,

(17) $$A_{x}=l\cdot w$$

(18) $$A_{y}=l\cdot s$$

Rearranging Equation (17):

(19) $$l=\;\frac{\;A_{x}}{w}$$

Put the value of *l* from Equation (19) in Equation (18):

(20) $$A_{y}=\frac{\;A_{x}}{w}\cdot s$$

(21) $$\frac{A_{y}}{\;A_{x}}=\frac{s}{w}=c$$

or:

(22) $$A_{y}=c\cdot A_{x}$$

where:

$$c=\;\frac{s}{w}$$

### 3.2. Value of w

The width of all different sides of the outer surfaces of stents was measured at 200 spots and then all lengths were averaged according to the weighted percentage of the particular part, as shown in Equation (23).

(23) $$w=\frac{\sum_{i=1}^{n}a_{i}w_{i}}{\sum_{i=1}^{n}w_{i}},$$

where *w_i_* is the individual width and *a_i_* is the weight of that width. *n* is the number of measurements taken.

## 4. Algorithm of the New Model for Corrosion Rate Calculation

**Figure 4:**
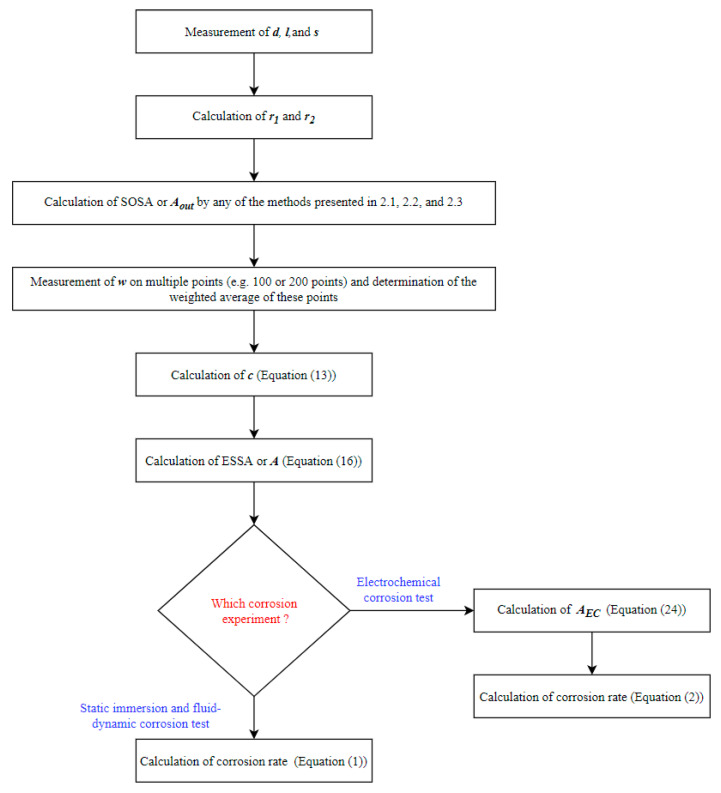
Schematic diagram of the algorithm to apply the surface area model for corrosion rate calculation.

Figure 4 presents the schematic flow diagram showing all the steps to calculate the ESSA or *A* and its application for the corrosion rate calculations in different experiments. After the calculation of *A*, the corrosion rates of stents can be calculated using Equation (1) for the static immersion test and fluid-dynamic test. Whereas, Equation (2) can be used for the calculation of the electrochemical corrosion rate. The whole length of the stent is not used in electrochemical corrosion tests. Therefore, *A_EC_* should be used instead of *A* (Equation (24)).

(24) $$A_{EC}=\frac{l_{EC}}{l}A$$

where *A_EC_* is the stent surface area used in the electrochemical corrosion test, *l* is the total stent length, *l_EC_* is the length of the stent used in the electrochemical corrosion test, and *A* is the ESSA.

## 5. Validation of the New Mathematical Model

The obtained results were validated by the following protocol.

**Figure 5:**
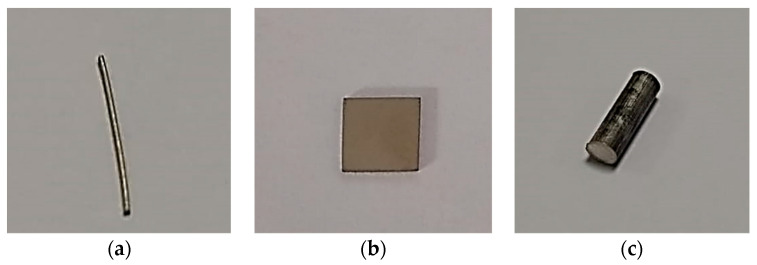
Samples with simpler geometry: (**a**) Mg alloy wire, (**b**) stainless steel, and (**c**) Mg alloy AZ31.

1. Surface area calculation of the simpler samples by micro-CT [23].
  - Samples with a different geometry and material were selected (Figure 5).
  - Micro-CT needs a reference volume for the surface area calculation of samples. Two different methods were used for the volume calculation of samples.
  - Micro-CT measurement of samples was conducted.
  - Reference volume by mass was used for the surface area calculation of samples.
2. Surface area calculation of stents by micro-CT.
  - Volume determination of stents by mass.
  - Micro-CT measurement of Mg alloy stent samples was conducted.
  - Surface area calculation using the reference volume by mass.
  - Comparison of the results by model and micro-CT.

### Micro-CT Measurement

**Figure 6:**
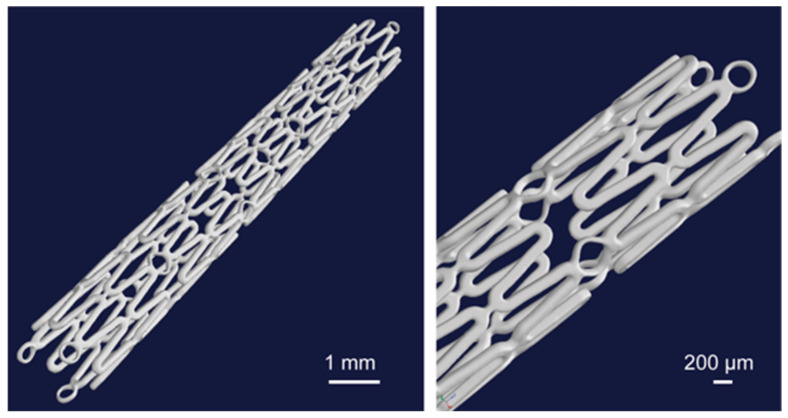
3D representation of the whole stent volume from the micro-CT investigation [23].

All the micro-CT measurements were conducted on a pre-clinical micro-CT Scanner SCANCO Medical viva CT 75 (SCANCO Medical AG, 8306 Bruettisellen, Switzerland). The maximum X-ray energy used was 70 keV at 114 µA. The voxel resolution was 20 µm using 2000 scan positions, with an exposure time per position of 350 ms. The volume and surface quantification was performed with the Scanco evaluation software (Version 6.5, SCANCO Medical AG, 8306 Bruettisellen, Switzerland). Figure 6 shows the reconstructed images of the Mg alloy stent after micro-CT.

## 6. Results

The structure of the used resoloy (RS) (Mg alloy) stents can be seen in the 3D reconstructed images (Figure 6). The symbol RS is used for simplicity instead of the full name of the used resoloy stents for the presentation of the results. The outer radius (r_1_) and inner radius (r_2_) were measured at 1 mm and 0.875 mm, respectively. The strut thickness of all the samples was 125 µm. Table 1 shows the Stent outer surface area and the Entire stent surface area calculations based on the derived equation. The average width of the upper surface was 0.121 mm. The obtained s and w gave the value of c as 1.03. The maximum calculated SOSA and ESSA were 33.62% and 112.77 mm^2^, respectively (RS-I, RS-II, and RS-IV).

Table 2 shows the comparison of the ESSA calculated from the derived equation and the micro-CT. The maximum difference between the calculated value and the micro-CT value is 12.92% (RS-IV). The maximum difference in the calculated reference volume for micro-CT and the nearest volume value used as the threshold was 0.04 mm^3^ in the case of RS-II.

The volume obtained by the density formula was highly dependent upon the mass of the stent. All seven samples have two mass values, therefore we obtained two values for the reference volumes for all the samples so far.

However, the micro-CT measurements directly depend on the geometry but are not influenced by mass. Therefore, we obtained variable values of micro-CT calculated surface areas for all samples.

**Figure 7:**
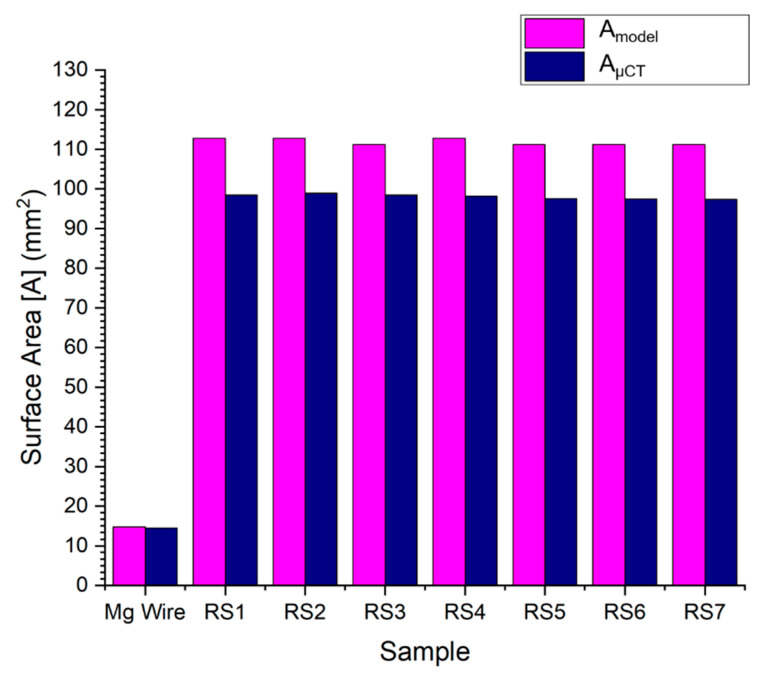
Results of the entire stent surface area, calculated by the developed model and micro-CT.

Figure 7 shows the surface areas calculated by equation and micro-CT measurement. The bar chart shows the consistent results of the samples.

## 7. Discussion

To evaluate the degradation behavior of metallic stents, corrosion experiments are used. For the corrosion rate calculation with different methods—for example, static immersion test, fluid dynamic test, and electrochemical corrosion test—we need the exposed surface area of the sample. For the standard geometrical shape, it is simple but for the samples that have complex geometry, like stents, it is very complex. For this purpose, innovative approaches are required to determine the entire stent surface area. In our previous work, we presented the application of micro-CT for the determination of the entire stent surface area. Since micro-CT has already been applied for the surface area calculations of different implant materials and biological objects, the reference volume used in the determination of surface area with the micro-CT was calculated by density formula, as previously described in [23].

However, micro-CT is a time-consuming and expensive method and is not a standard characterization method in most research labs. As an alternative non-destructive approach to estimate the surface area of stents, a novel mathematical model has been developed and validated with micro-CT data. This is the first ever model to calculate the ESSA. This could offer the possibility for a faster, reliable stent characterization. The equipment which is needed for the introduced mathematical model will be an SEM, and most labs have access to it.

In our previous study [23], we presented the surface area calculations of different standard geometrical shapes (wire, cylindrical rod, rectangular, and square) and materials. In those results, Mg alloy wire gave the minimum difference in the calculated surface area values from the standard model and the micro-CT. Therefore, in the present work, we used that result as a reference to the current results of Mg alloy stents.

By using the proposed model, corrosion tests on stents can be conducted easily. The exposed surface area of stents is inversely proportional to the corrosion rate. Based on the difference we got in ESSA calculation by the newly developed model and micro-CT, the corrosion rate calculated by using ESSA obtained from the model will be 12.34% (±0.46%), less than that ESSA obtained by micro-CT.

This study showed good results for Mg alloy samples with the developed model. However, surface area calculations of other stent samples including different materials and designs are necessary. The surface area calculations of the drug eluting stents (DES) and coated stents are here.

A possible reason for the smaller surface area values from micro-CT could be that the visualization of the stent wires is influenced by X-ray beam hardening at the metal surface, which could result in an underexposed stent wire volume. Another possible reason is that the SOSA used in the calculation of ESSA is based on the mass of the sample. The study by Karoly et al. 2013 [21] discussed that the manual and automatic stent scanning methods to calculate SOSA are more accurate. The application of these methods may further reduce this difference.

A limitation of the developed model is the use of the average width of the upper surface. This is also very important and it highly depends on the number of lengths taken for getting an average. In the presented study, 200 measurements were undertaken. However, we observed that the result was similar for 100 measurements. In future studies, and with other stent designs, the number of measurements for the weighted average may be standardized.

## 8. Conclusions

Stenting is a fast-growing research field due to it being less invasive, its live-saving capabilities, and it being less risky than coronary artery bypass grafting (CABG). Despite huge advancements, still significant restenosis rates and undesirable clinical outcomes make this field still very open for advancements. One important step towards the further development of this technology is to use stents for all experiments rather than using simpler samples of the stent material. Therefore, we have introduced a novel mathematical model for the calculation of the entire stent surface area. The model was used for the calculation of the surface area of Mg alloy stents, and the results have been validated with micro-CT measurements. The difference in the results from the model and micro-CT can be minimized using the manual and automatic stent scanning methods for the calculation of the stent outer surface area and avoiding beam-hardening effects for micro-CT—for example, using synchrotron sources for micro-computed tomography (SR micro-CT).

## Figures and Tables

**Table 1:** Surface area calculation using the developed model.

S.No.	Sample	Average Width of Upper Surface	Surface Co-Efficient	Stent Outer Surface Area (SOSA)	Entire Stent Surface Area (ESSA)
		w (mm)	c	A_out_ (%)	A (mm^2^)
1	RS-I	0.121	1.03	33.62	112.77
2	RS-II	0.121	1.03	33.62	112.77
3	RS-III	0.121	1.03	33.14	111.18
4	RS-IV	0.121	1.03	33.62	112.77
5	RS-V	0.121	1.03	33.14	111.18
6	RS-VI	0.121	1.03	33.14	111.18
7	RS-VII	0.121	1.03	33.14	111.18

RS—Resoloy (Mg alloy) stent.

**Table 2:** Validation of the calculated surface area with micro-CT measurement.

S.No.	Sample	Volume		ESSA		Difference
		Calculated	Micro-CT	Calculated	Micro-CT	-
		V_Model_ (mm^3^)	V_µCT_ (mm^3^)	A_Model_ (mm^2^)	A_µCT_ (mm^2^)	%
1	Mg alloy wire	1.59	1.59	14.77	14.45	2.17
2	RS-I	3.70	3.67	112.77	98.49	12.66
3	RS-II	3.70	3.66	112.77	98.95	12.26
4	RS-III	3.65	3.63	111.18	98.49	11.42
5	RS-IV	3.70	3.70	112.77	98.20	12.92
6	RS-V	3.65	3.65	111.18	97.53	12.28
7	RS-VI	3.65	3.63	111.18	97.41	12.39
8	RS-VII	3.65	3.63	111.18	97.34	12.45

## References

[1] Serruys P.W., Morice M.-C., Kappetein A.P., Colombo A., Holmes D.R., Mack M.J., Ståhle E., Feldman T.E., Brand M.V.D., Bass E.J. (2009). Percutaneous Coronary Intervention versus Coronary-Artery Bypass Grafting for Severe Coronary Artery Disease. N. Engl. J. Med..

[2] Schmidt T., Abbott J. (2018). Coronary Stents: History, Design, and Construction. J. Clin. Med..

[3] Silvain J., Cayla G., Collet J.-P., Montalescot G., Fargeot C. (2014). Coronary Stents: 30 Years of Medical Progress. Medecine/Sciences.

[4] MacNeill B.D., Jang I.K., Wong P. (2005). Coronary Stents. Clinical, Interventional and Investigational Thrombocardiology.

[5] Boccalini S. (2019). CT Imaging of the Thoracic Aorta: Size Assessment and Follow-Up after Surgical and Endovascular Intervention.

[6] Costa F., van Klaveren D., James S., Heg D., Räber L., Feres F., Pilgrim T., Hong M.K., Kim H.S., Colombo A. (2017). Derivation and Validation of the Predicting Bleeding Complications in Patients Undergoing Stent Implantation and Subsequent Dual Antiplatelet Therapy (PRECISE-DAPT) Score: A Pooled Analysis of Individual-Patient Datasets from Clinical Trials. Lancet.

[7] Mohapatra A., Saadeddin Z., Bertges D.J., Madigan M.C., Al-Khoury G.E., Makaroun M.S., Eslami M.H. (2020). Nationwide trends in drug-coated balloon and drug-eluting stent utilization in the femoropopliteal arteries. J. Vasc. Surg..

[8] Lei Y., Chen X., Li Z., Zhang L., Sun W., Li L., Tang F. (2020). A New Process for Customized Patient-Specific Aortic Stent Graft Using 3D Printing Technique. Med. Eng. Phys..

[9] Saito Y., Grubman D., Cristea E., Lansky A. (2020). The Firehawk Stent: A Review of a Novel Abluminal Groove-Filled Biodegradable Polymer Sirolimus-Eluting Stent. Cardiol. Rev..

[10] Majewska P., Oledzka E., Sobczak M. (2020). Overview of the latest developments in the field of drug-eluting stent technology. Biomater. Sci..

[11] ASTM (2010). ASTM G102-89 Standard Practice for Calculation of Corrosion Rates and Related Information from Electrochemical Measurements.

[12] Wong L.L., Martin S.I., Rebak R.B., Columbia B., Wong Sue I Martin Raúl B Rebak L.L. Methods to Calculate Corrosion Rates for Alloy 22 from Polarization Resistance Experiments. Proceedings of the ASME Pressure Vessels and Piping Conference.

[13] Gastaldi D., Sassi V., Petrini L., Vedani M., Trasatti S., Migliavacca F. (2011). Continuum Damage Model for Bioresorbable Magnesium Alloy Devices—Application to Coronary Stents. J. Mech. Behav. Biomed. Mater..

[14] Grogan J.A., Leen S.B., McHugh P.E. (2014). A Physical Corrosion Model for Bioabsorbable Metal Stents. Acta Biomater..

[15] Grogan J.A., Leen S.B., McHugh P.E. (2014). Computational Micromechanics of Bioabsorbable Magnesium Stents. J. Mech. Behav. Biomed. Mater..

[16] Ma Z.M., Dong H.Y., Qi M. (2012). A Study on Fatigue Test for Cardiovascular Stent. Appl. Mech. Mater..

[17] Pelton A.R., Schroeder V., Mitchell M.R., Gong X.-Y., Barney M., Robertson S.W. (2008). Fatigue and Durability of Nitinol Stents. J. Mech. Behav. Biomed. Mater..

[18] Hertel M., Laule M., Zinelis S., Imiolczyk S.M., Mueller W.-D. (2016). Electrochemical Characterization of Vascular Bare-Metal Stents. A Novel Approach Modifying the Mini-Cell System. SDRP J. Biomed. Eng..

[19] Hertel M. (2012). Elektrochemische Charakterisierung von Stents Mit Hilfe Des Adaptierten Mini-Cell-Systems (MCS)–Teil II. Ph.D. Thesis.

[20] Károly D., Asztalos L., Fazakas É. Corrosion Behaviour Of Overlapping Coronary Artery Stents. Proceedings of the 34th Danubia-Adria Symposium on Advances in Experimental Mechanics.

[21] Károly D., Kovács M., Terdik A.A., Bognár E. (2013). Investigation of Metallic Surface Area of Coronary Stents. Biomech. Hung..

[22] Kovács M., Károly D., Dévényi L. (2015). Comparing Two Examination Methods for Measuring Metal to Artery Ratio of Coronary Stents. Mater. Sci. Forum.

[23] Saqib M., Bernhardt R., Kästner M., Beshchasna N., Cuniberti G., Opitz J. Application of ΜCT for the Determination of Total Surface Area of Stents. Proceedings of the 7th E-Health and Bioengineering Conference, EHB 2019.

[24] ISO (2012). ISO 25539-2: Cardiovascular Implants-Endovascular Devices—Part 2: Vascular Stents.

